# Supplementary material for: The causal role of circulating inflammatory markers in osteoporosis: a bidirectional Mendelian randomized study
Source: Front Immunol. 2024 Jul 18;15:1412298. doi: 10.3389/fimmu.2024.1412298 (PMC11291241; doi:10.3389/fimmu.2024.1412298)
Supplement: Supplementary file 1 [file DataSheet_1.zip › supplementary material/CIM-OSTEOPOROSIS /GCST90274834.csv_forest.pdf]

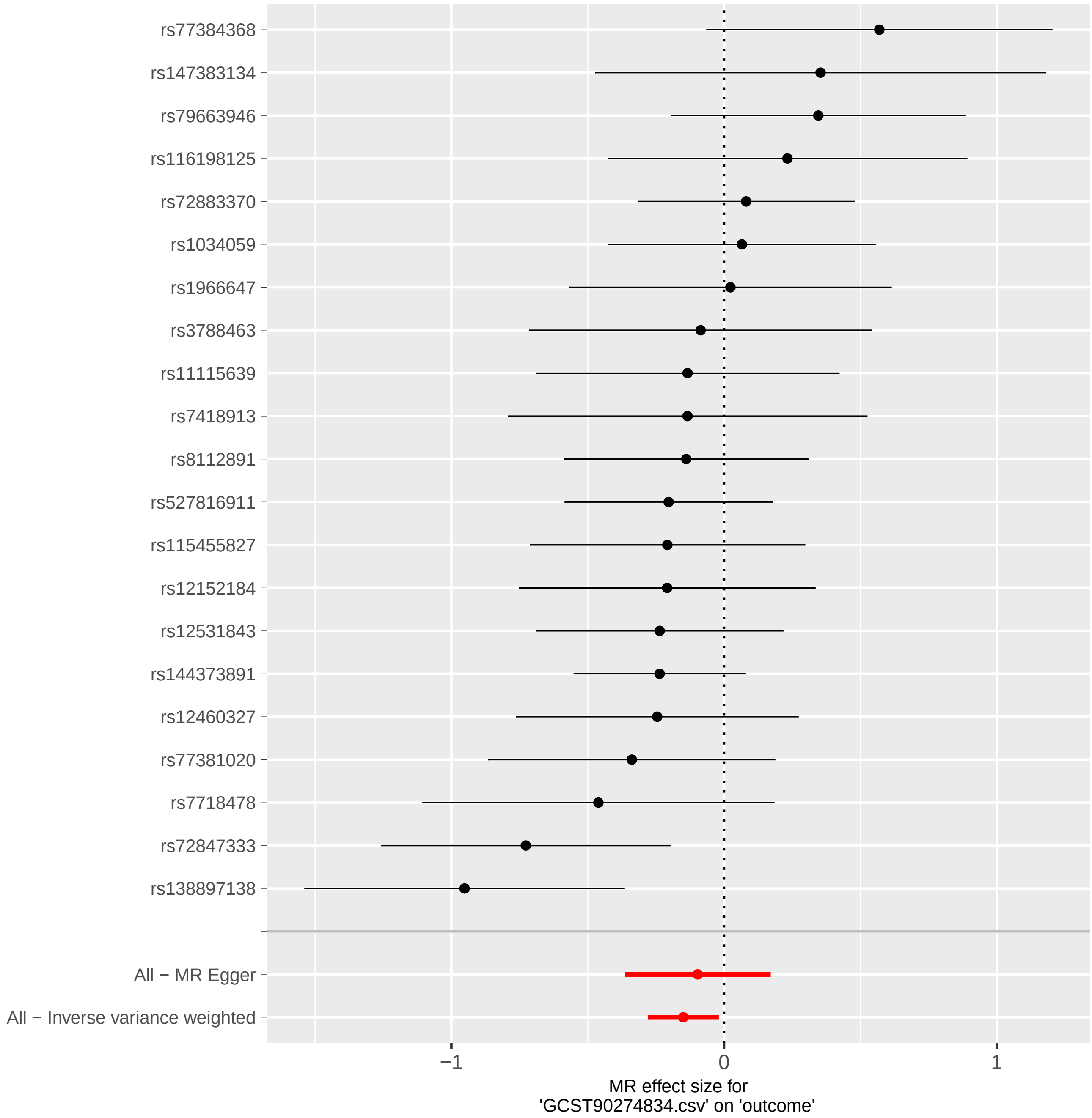
rs77384368
rs147383134
rs79663946
rs116198125
rs72883370
rs1034059
rs1966647
rs3788463
rs11115639
rs7418913
rs8112891
rs527816911
rs115455827
rs12152184
rs12531843
rs144373891
rs12460327
rs77381020
rs7718478
rs72847333
rs138897138
All − MR Egger
All − Inverse variance weighted
−1
0
1
MR effect size for
'GCST90274834.csv' on 'outcome'
